# Meal Timing and Depression Among Chinese Children and Adolescents: Cross-Sectional Study

**DOI:** 10.2196/54275

**Published:** 2024-10-23

**Authors:** Huilun Li, Zhaohui Lu, Erliang Zhang, Jie Zhang, Shuheng Cui, Masaki Takahashi, Mi Xiang

**Affiliations:** 1 Hainan Branch, Shanghai Children’s Medical Center, School of Medicine, Shanghai Jiao Tong University, Sanya, China; 2 School of Public Health, Shanghai Jiao Tong University, Shanghai, China; 3 Institute for Liberal Arts, Tokyo Institute of Technology, Tokyo, Japan

**Keywords:** mental health, meal timing, chrononutrition, depression, mhealth, meal time, children, adolescent, cross-sectional study, China, schedule of meal, metabolic disorder, correlation, survey, breakfast skipping, food intake, daily eating, analysis, logistic regression

## Abstract

**Background:**

Depression in children and adolescents is a rising concern in China. Dietary behavior is a critical determinant of mental health. Meal timing, or the schedule of meal consumption, has been related to several metabolic disorders. However, the effect of meal timing on mental health is scarce, particularly in children and adolescents who are in a critical period of physical and psychological development.

**Objective:**

This research examined the relationship between meal timing and depression in children and adolescents in China.

**Methods:**

Children and adolescents from grades 1 to 9 were recruited from 16 districts in Shanghai, China, from January 3 to January 21, 2020. Ten schools attended the study. A survey was distributed to the students and their parents to collect demographic and health-related information. Depression was measured by the Children’s Depression Inventory-Short Form. Breakfast consumption was analyzed as a binary outcome. Participants were defined as breakfast consumers if they never skipped breakfast during the week. They were otherwise defined as breakfast skippers if they skipped breakfast at least once per week. A similar categorization was applied to analyze food intake proximal to bed. Daily eating windows were calculated using the last food intake time frame—the first food intake time frame. Participants were classified into eating window groups of less than 10 hours, 10-12 hours, and more than 12 hours. A logistic regression model was used to compute the odds ratio (OR) and 95% CI.

**Results:**

A total of 6874 participants were included in the analysis. Participants who skipped breakfast were associated with a 2.70 times higher occurrence of depression (OR 2.70, 95% CI 2.24‐3.26; *P*<.001). The prevalence of depression was 1.28 times higher in participants who ate before bed than in those who never ate before bed (OR 1.28, 95% CI 1.08‐1.50; *P*<.001). The occurrence of depression was 1.37 times higher if the eating time window was shorter than 10 hours (OR 1.37, 95% CI 1.08‐1.73; *P*=.009) and 1.23 times higher if the eating time window was longer than 12 hours (OR 1.23, 95% CI 1.01‐1.50; *P*=.004). The lowest occurrence of depression was observed at 11.5 hours. Subgroup analysis showed that such relationships remained significant in adolescents aged 10 years or older. In children, only skipping breakfast was associated with a higher odds of depression (OR 2.77, 95% CI 1.94‐3.96; *P*<.001).

**Conclusions:**

Breakfast skipping and eating before bed significantly increase the occurrence of depression. The optimal daily eating window to lower the occurrence of depression is 11.5 hours in children and adolescents. Daily eating windows longer than 12 hours or shorter than 10 hours are associated with an elevated occurrence of depression. Current findings advocate evidence-based dietary strategies to prevent and treat depression in children and adolescents.

## Introduction

Mental disorders are a major contributor to disability-adjusted life-years worldwide, accounting for 4.9% of global disability-adjusted life-years and 125.3 million cases of years lived with disabilities [1]. The accelerated socioeconomic development in China has led to a substantial elevation in mental illness incidence [2]. There has been a growing concern about childhood and adolescents’ psychological health since more than half of the first mental illness onset occurs in adolescence [3]. As a key population segment, mental disorders, depression, in particular, have become alarmingly ubiquitous in Chinese children and adolescents [4]. Psychological health is particularly significant during childhood and adolescence. This pivotal period is characterized by rapid growth, hormonal changes, and lifelong behavioral establishment, laying the foundation for overall health and well-being in adulthood [5].

Depression is one of the most prevalent mental illnesses, with approximately 25% of teenagers experiencing depression episodes by the age of 19 years [6]. The global prevalence of depressive symptoms reaches as high as 21.3% in children and adolescents [6]. In China, the rate is 17.2% in primary school students [7]. However, distinct challenges are faced by individuals with depression in China. The diagnostic and treatment rates are low in China, possibly due to limited health services and stigmatization of the disease [8]. On the other hand, limited treatment options with compromised effectiveness are a major obstacle to depression management [8].

Depressive symptoms in childhood and adolescence commonly persist in adulthood, resulting in lifelong burdens for the individual [9]. More noticeably, severe depression is one of the most prominent risk factors for suicide, resulting in devastating consequences [10]. The increased burden of pediatric depression on the health care system and the unique challenges faced by Chinese individuals are calling for urgent action. Thus, diet as one modifiable while prominent driving factor of depression in children and adolescents merits scrutiny [11].

Dietary behavior is one of the most prominent lifestyle determinants of human health. Diet and nutrition studies have been centered on qualitative and quantitative aspects for decades. In recent years, the temporal characteristics of meal consumption have become an emerging field of research. Unhealthy dietary behavior may lead to desynchronized circadian rhythms, altering several metabolic and hormonal responses [12,13]. Meal timing, defined as the timing of each meal and the duration of the first meal to the last meal consumption in a day, has been reported to exert a notable effect on human circadian rhythms [14]. Coinciding meal intake with circadian rhythm, a rising field of research known as chrononutrition, may optimize general health in human beings [15,16].

Irregularity of meal timing has been shown to increase the risk of weight gain [17], metabolic biomarkers [18], and unfavorable cardiometabolic outcomes [19]. In children and adolescents, meal timing has been associated with insulin resistance [20]. Irregular meal intake time may desynchronize the central and peripheral clocks by affecting peripheral organs and glucose and lipid metabolism, which disrupts circadian rhythms [14,21]. Circadian misalignment, as a result of irregular food consumption, is associated with several mental illnesses, such as mood disorders, anxiety, and depression [22]. A previous study has revealed a converse relationship between irregular meal timing and subjective mental health [23]. Although research examining the association between meal timing and mental health in children and adolescents is scarce, growing speculations on the effect of meal timing on mental conditions, such as depression and anxiety, have been proposed [24].

Another aspect of meal timing is the daily eating window, the span of all food consumption in a day, which is linked to the risk of metabolic diseases and certain cancers [25]. The food intake window may modulate neurochemistry and neuronal activity, suggesting the potential contribution of the eating window to mental health. Nevertheless, the effect of the daily feeding window on mental health outcomes remains controversial [22]. Longer fasting night intervals have been related to lower systemic inflammation and better metabolic biomarkers [13,18], potentially benefiting mental health. In contrast, researchers have also found an association between limiting eating time and a higher risk of cognitive impairment in older adults in China [26]. Therefore, the optimal daily intake window is yet to be explored.

Diet is a prominent yet highly modifiable factor that determines human health. Dietary habits are even more critical in childhood and adolescence as they shape lifelong lifestyles. Unlike changing meal patterns and intakes, adjusting meal timing is highly feasible and generalizable in children and adolescents. Understanding the temporal aspect of dietary behavior in this critical period sheds light on the prevention and management of various mental health issues and warrants in-depth discussion. However, there is a paucity of evidence about the influence of meal timing on depression in children and adolescents. In addition, meal-timing interventions for children and adolescents are very limited due to uncertainties in optimal timing and duration and potential adverse effects on growth. Therefore, this study attempts to explore the intricate relationship between meal timing and depression in children and adolescents.

## Methods

### Study Design

This cross-sectional study recruited children and adolescents in Shanghai, China. Multistage clustering sampling was used to sample school-aged children and adolescents from 16 districts in Shanghai from January 3 to January 21, 2020. Seven districts agreed to participate in the study. One to 2 schools in each of the 7 districts were randomly selected and contacted for possible research collaboration. Ten schools agreed to participate, covering 7 districts in total. A web-based survey was distributed by the designated teachers to school-aged children and adolescents via WeChat. Participants completed a web-based questionnaire via the Wenjuanxing platform. Our research team trained the designated teachers at each school regarding research requirements, application, and context before the study. The designated teachers and researchers explained and provided assistance when needed throughout the study. The survey contained 2 separate parts for the students and parents. The parental questionnaire collected information about the family and the parents themselves, such as the parents’ education level and family income. The students’ survey collected demographic characteristics, mental health, physical health, and dietary habits of the students. The designated teachers and researchers verbally explained the study and consent procedure to the children and adolescents before distributing the written informed consent to their parents. Parents were allowed and encouraged to assist their children, particularly young children, in answering uncertain questions to avoid potential recall bias since young children might have comprehension difficulties or uncertainties regarding the questions. The survey also provided a prompt at the beginning to remind the parents to assist younger participants in filling in more accurate answers.

### Ethical Considerations

Consent was obtained from all participants before the study. Ethical approval was obtained from the Shanghai Jiao Tong University School of Medicine (SJUPN-201813).

### Participants

**Figure 1.**
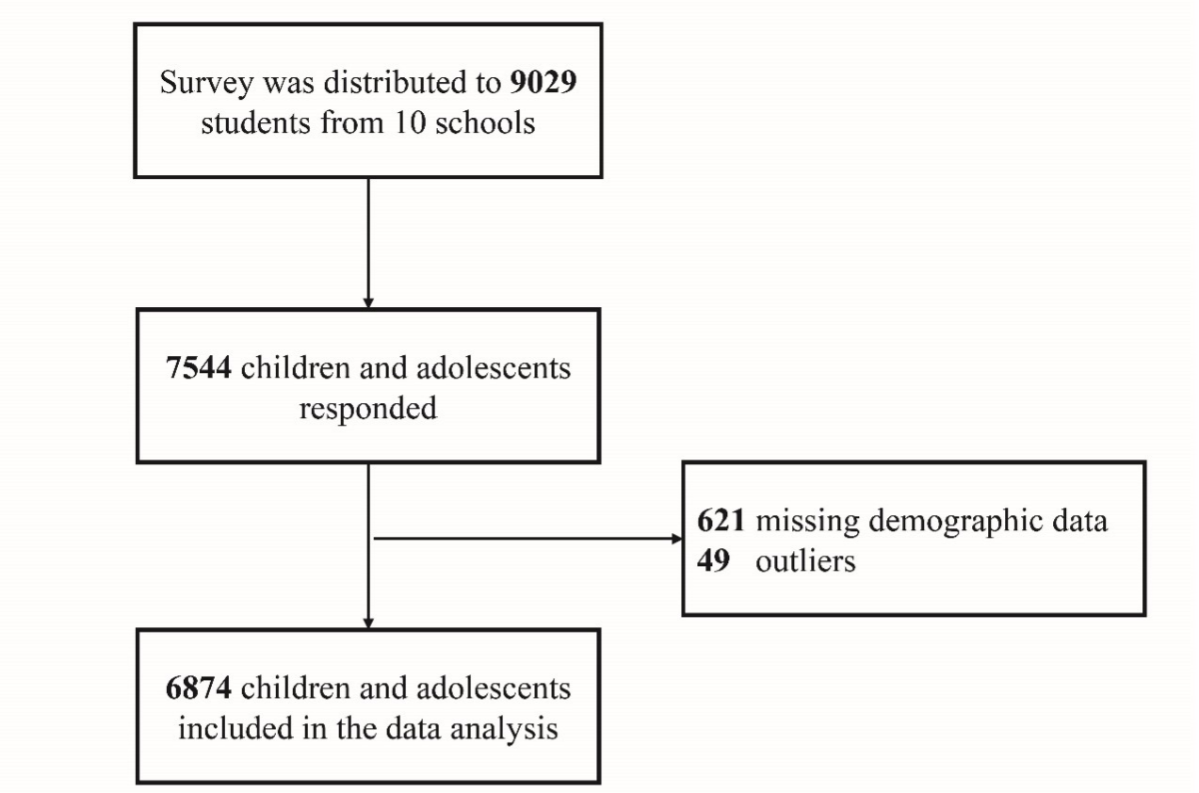
Flow diagram of selecting the study sample from 10 participating schools in China.

Children and adolescents from grades 1 to 9 were eligible for the study. All students and parents in the participating 10 schools were invited to attend the study. Individuals with any severe psychiatric conditions or physical health issues that impact the accuracy of the reporting, such as severe metabolic disorders, dementia, retardation, or any condition that disables them from filling the questionnaire, were excluded. A total of 9029 surveys were distributed to participating students. By the end of the collection, 7544 answers were received, corresponding to an 83.6% response rate. After excluding 621 demographic missing data (mostly from parental answers) and 49 outliers, a total of 6874 individuals were included in this analysis (Figure 1).

### Depression

Depression was measured by the Children’s Depression Inventory-Short Form [27]. The questionnaire is widely used and showed high internal consistency (Cronbach *α*=.75) in the study of Chinese children. In this research, depressive symptoms were defined as a score greater than or equal to 7 [28].

### Meal Timing

The daily eating window was assessed based on multiple questions, including meal timing, whether they skipped breakfast, and whether they consumed food before bed. The eating time span was calculated as the last food intake time frame—the first food intake time frame. Previous studies showed an eating window of less than 10 hours may impair cognitive function [26], while an eating window of less than 12 hours showed beneficial outcomes [29]. Therefore, eating window time was grouped into less than 10 hours, 10-12 hours, and more than 12 hours, with 10-12 hours being the reference group in logistic regression analysis.

Breakfast frequency was evaluated using one question: “How often do you consume breakfast in a week?” Answers ranged from “none” to “every day.” Participants who skipped breakfast at least once per week were categorized into the breakfast-skipper group [30]. Participants who ingested breakfast every day of the week were categorized as the breakfast consumer group. Since skipping breakfast has been reported to increase depression risks [31], the breakfast consumer group was the reference group in our logistic regression analysis.

Food intake proximal to bedtime was determined based on one question: “How often do you usually consume food 2 hours before sleep in a week?” Participants who answered one or more times were classified as the eating-before-bed group. Delayed eating time has been related to a higher risk of mental disorders in college students [32]. Thus, participants who never eat before bed were the reference group in our logistic regression analysis.

### Covariates

Possible covariates, such as age, sex, BMI, family education level, and family income, were assessed using the demographic questionnaire of the survey. Age was reported as a continuous variable. BMI was calculated using the reported height and weight. Participants chose from <US $100,000, US $100,000‐$200,000, US $200,000‐$400,000, and >US $400,000, or refused to answer when answering their family income. Answers for education level contained junior high school and below, high school, college or university, and master or higher. We also adjusted total caloric intake as one possible covariate since calorie restriction has been related to depression [33]. Dietary caloric intake was assessed using a validated Chinese food frequency questionnaire for children and adolescents [34].

### Statistical Analysis

Continuous variables were assessed for normality using the Shapiro-Wilk test. Descriptive statistics presented normally distributed continuous variables in median and IQR (median, Q1-Q3) and categorical variables in frequencies and percent distribution (n%). Nonnormally distributed continuous variables are presented in medians and IQRs. Intergroup comparison of the continuous and categorical variables was made using the Pearson chi-square test or Student *t* test when appropriate. The logistic regression model was used to compute the dds ratio (OR) and 95% CI, adjusting covariates. When examining a single aspect of meal timing, other aspects were also adjusted as potential confounders since they were reported to influence health outcomes. Restricted cubic splines were used to plot and visualize the relation between eating window time and depression. Adolescence is a period of dramatic physical and psychological change and maturation. Dietary behavior may exert distinct effects on children and adolescents due to physiological and psychological differences. Thus, we performed a subgroup logistic regression analysis separately on children and adolescents. Children and adolescents were distinguished by the age of 10 years, according to the definition by the World Health Organization [35]. The “rms” package in R software (The R Foundation) was applied. A *P* value of less than .05 was considered significant. All statistical analyses were 2-tailed tests and were performed using R (version 4.2.2; The R Foundation).

## Results

### Study Population

A total of 6874 participants were included in the study. The demographic characteristics of the study participants are summarized in Table 1. Participants were aged 5-17 years, with a median age of 9 years. The median BMI was 17.7. A higher proportion of males (n=3558, 51.8%) than females (n=3316, 48.2%) was observed. Most participants (n=2208, 32.1%) had a family income of CNY 100,000-200,000 (US $14,193-$28,385). Most parents in the study population reported having a college or university degree (n=3989, 58.0% father and n=4143, 60.3% mother).

A total of 88.8% (n=6106) of the study sample ate breakfast every day in a week (Table 2). Most children and adolescents ingested food 2 hours before bed at least once a week (n=4498, 65.4%). The median eating window was 11.5 hours.

**Table 1.** Demographic characteristics of study participants according to depression incidence (N=6874).

Variables	Overall	Depression^a^		*P* value
		No	Yes	
Sample size, n (%)^b^	6874 (100)	6012 (87.5)	862 (12.5)	
**Sex, n (%)**				.12
Girls	3316 (48.2)	2879 (47.9)	437 (50.7)	
Boys	3558 (51.8)	3133 (52.1)	425 (49.3)	
Age (years), median (Q_1_, Q_3_)^c^	9 (8, 12)	9 (8, 12)	11 (9, 13)	<.001
BMI (kg/m^2^), median (Q_1_, Q_3_)	17.7 (15.5, 20.8)	17.5 (15.4, 20.6)	19.0 (16.6, 22.3)	<.001
**Parental education, n%**				
**Father**				<.001
Middle school or below	801 (11.7)	668 (11.1)	133 (15.4)	
High school	1599 (23.3)	1367 (22.7)	232 (26.9)	
University or college	3989 (58.0)	3551 (59.1)	438 (50.8)	
Master’s degree or higher	456 (6.6)	404 (6.7)	52 (6.0)	
Unknown	29 (0.4)	22 (0.4)	7 (0.8)	
**Mother**				<.001
Middle school or below	949 (13.8)	782 (13.0)	167 (19.4)	
High school	1481 (21.5)	1267 (21.1)	214 (24.8)	
University or college	4143 (60.3)	3696 (61.5)	447 (51.9)	
Master’s degree or higher	267 (3.9)	240 (4.0)	27 (3.1)	
Unknown	34 (0.5)	27 (0.4)	7 (0.8)	
**Family income (CNY), n (%)**				<.001
<100,000	976 (14.2)	778 (12.9)	198 (23.0)	
100,000-200,000	2208 (32.1)	1937 (32.2)	271 (31.4)	
>200,00-400,000	2110 (30.7)	1876 (31.2)	234 (27.1)	
>400,000	911 (13.3)	821 (13.7)	90 (10.4)	
Withhold	669 (9.7)	600 (10.0)	69 (8.0)	
**Calorie intake (kcal), median (Q**_**1**_ **, Q**_**3**_ **)**	2415(1619, 3425)	2429 (1636, 3424)	2309 (1403, 3454)	.06

a Depression: participants are categorized into the depression group if scored greater than or equal to 7 using the Children’s Depression Inventory-Short Form

b N (%): categorical variables are displayed in frequencies and percent distribution (n%).

c Median (Q1, Q3): non-normally distributed continuous variables are presented in medians and IQR.

**Table 2.** Frequencies of breakfast consumption, eating before bed, and daily eating window time variables according to depression incidence.

Variables	Overall	Depression^a^		*P* value
		No	Yes	
**Breakfast consumption** ^**b**^ **, n (%)**				
Consuming breakfast	6106 (88.8)	5473 (91.0)	633 (73.4)	
Skipping breakfast	768 (11.2)	539 (9.0)	229 (26.6)	
**Eating before bed** ^**c**^ **, n (%)**				<.001
No	2376 (34.6)	2124 (35.3)	252 (29.2)	
Yes	4498 (65.4)	3888 (64.7)	610 (70.8)	
Daily eating window (hours)^d^, median (Q_1_, Q_3_)	11.5 (10.5, 12.5)	11.5 (10.5, 11.5)	11.5 (10.5, 11.5)	.09

a Depression: participants are categorized into the depression group if scored greater than or equal to 7 using the Children’s Depression Inventory-Short Form.

b Breakfast consumption: participants who skipped breakfast at least once per week are categorized into the skipping breakfast group.

c Eating before bed: participants eat one or more times before bed are classified as the eating-before-bed group.

d Daily eating window: last food intake time frame—first food intake time frame.

### Evaluation Outcomes

The finding showed that 12.5% (n=862) of the study participants screened positive for depressive symptoms. The median age of the depression group is 11 years, older than that of the nondepressive group (9 years). A higher BMI was observed in the depressive group than in the nondepressive group (19.0 vs 17.5). More parents attended university or college in the nondepressive group than in the depressive group. There is a significantly higher rate of children and adolescents skipping breakfast in the depressive group than in the nondepressive group (n=229, 26.6% vs n=539, 9%). Participants with depressive symptoms reported a higher proportion of eating before bed (n=610, 70.8% vs n=3888, 64.7%).

Three models were used in the logistic regression analysis. Model 1 was the crude model. Model 2 adjusted for age, sex, BMI, parental education level, and family income. Model 3 was further adjusted for caloric intake and different aspects of meal timing. As shown in Table 3, the breakfast skipper group reported 3.67 times higher odds of depression than the breakfast consumer group (OR 3.67, 95% CI 3.08‐4.38; *P*<.001). The odds of depression remained significantly higher (OR 2.70, 95% CI 2.24‐3.26; *P*<.001) after adjusting for age, sex, BMI, parental education level, family income, caloric intake, eating before bed, and eating time window. The eating-before-bed group indicated 1.32 times higher incidence of depression than participants who did not consume any food before bed (OR 1.32, 95% CI 1.13‐1.55, *P*<.001). In model 3, the odds of depression were 1.28 times greater in the eating-before-bed group than in participants who never ate before bed (OR 1.28, 95% CI 1.08‐1.50, *P*=.004).

**Figure 2.**
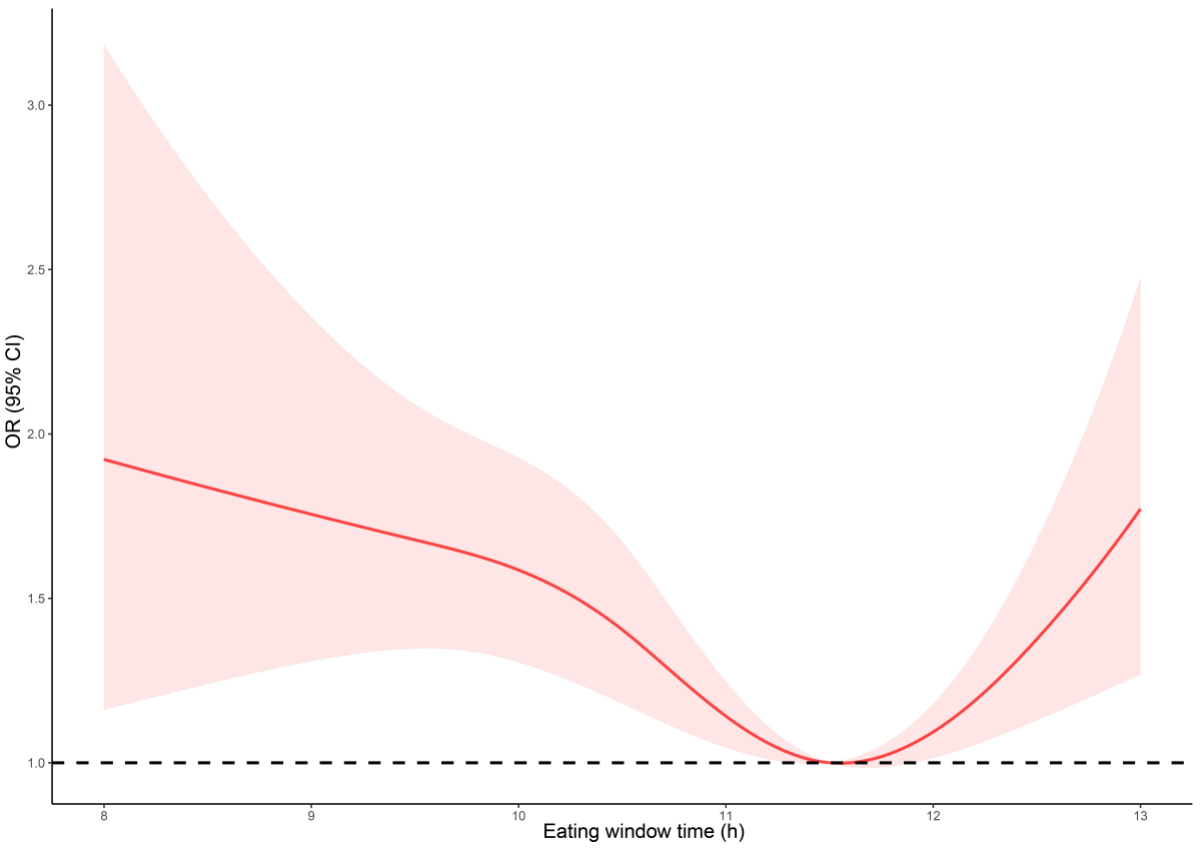
Restricted cubic splines plot of the relation between eating time window time and depression incidence. The optimal eating time window was between 11 and 12 h.

The occurrence of depression increased 1.47 times when the eating time window was less than 10 hours compared to the eating window at 10‐12 hours (OR 1.47, 95% CI 1.18‐1.83; *P*=.001). Similarly, participants who consumed food 12 hours more daily had a 1.8 times higher odds of depression than participants with an eating window at 10‐12 hours (OR 1.80, 95% CI 1.50‐2.17; *P*<.001). In model 3, controlling for age, sex, BMI, parental education level, family income, caloric intake, eating before bed, and breakfast eating, the depression incidence was 1.37 times higher if the eating time window was less than 10 hours (OR 1.37, 95% CI 1.08‐1.73; *P=.*009) and 1.23 times higher if the eating time window was more than 12 hours (OR 1.23, 95% CI 1.01‐1.50; *P*=.04). Results plotted by the restricted cubic splines illustrated the lowest odds of depression at 11.5 hours (Figure 2).

Subgroup analyses further stratified children and adolescent populations (Tables S1 and S2 in Multimedia Appendix 1). A significantly higher proportion of children consumed breakfast than adolescents (n=3276, 92.3% vs n=2830, 85.1%, *P*<.001). More children eat before bed than adolescents, with a marginal statistical difference (n=2361, 66.5% vs n=2137, 64.3%, *P*=.05). Adolescents had a significantly longer eating window than children (11.5 vs 10.5, *P*<.001).

Subgroup analysis demonstrates the relationships between children and adolescents separately (Table S2 in Multimedia Appendix 1). Skipping breakfast significantly increases the odds of depression in children (OR 2.77, 95% CI 1.94‐3.96; *P*<.001) and adolescents (OR 2.69, 95% CI 2.16‐3.35; *P*<.001). However, eating before bed and eating window time were not associated with the odds of depression in children. In adolescents, eating before bed increased the odds of depression by 1.38 times (OR 1.38, 95% CI 1.12‐1.69; *P*=.002). An eating window time less than or equal to 10 hours showed a significantly higher incidence of depression (OR 1.41, 95% CI 1.04‐1.91; *P*=.03) than an eating window time between 10 and 12 hours. Similarly, eating window time longer than or equal to 12 hours is associated with 1.27 times higher odds of depression (OR 1.26, 95% CI 1.01‐1.58; *P*=.04).

**Table 3.** Logistic regression analyses of breakfast consumption, eating before bed, and daily eating window in relation to depression incidence.

	Unadjusted modelOR (95%CI)	*P* value	Model 2^a^OR (95% CI)	*P* value	Model 3^b^OR (95% CI)	*P* value
**Breakfast consumption** ^**c**^						
Consuming breakfast	Reference		Reference		Reference	
Skipping breakfast	3.67 (3.08‐4.38)	<.001	2.83 (2.36‐3.40)	<.001	2.70 (2.24‐3.26)	<.001
**Eating before bed** ^**d**^						
No	Reference		Reference		Reference	
Yes	1.32 (1.13‐1.55)	<.001	1.40 (1.19‐1.64)	<.001	1.28 (1.08‐1.50)	.004
**Eating window time (hours)** ^**e**^						
10-12	Reference		Reference		Reference	
≤10	1.47 (1.18‐1.83)	.001	1.49 (1.19‐1.88)	<.001	1.37 (1.08‐1.73)	.009
≥12	1.80 (1.50‐2.17)	<.001	1.29 (1.06‐1.57)	.01	1.23 (1.01‐1.50)	.04

a Model 2 adjusted for age, sex, BMI, parental education level, and family income.

b Model 3 adjusted for caloric intake and other aspects of meal timing.

c Breakfast consumption: participants who skipped breakfast at least once per week are categorized into the skipping breakfast group.

d Eating before bed: participants who eat one or more times before bed are classified as the eating-before-bed group.

e Daily eating window: last food intake time frame – first food intake time frame

## Discussion

### Principal Findings

There is a growing body of literature evidence of the relationship between dietary behavior and depression in younger and older adults [31,32,36,37]. However, no study has explored meal timing and depression in children and adolescents to our knowledge. Understanding the impact of meal timing on depression is essential for developing effective strategies to promote optimal health in children and adolescents. We found skipping breakfast, eating before bed, and prolonged or restricted daily eating windows are associated with increased odds of depression in children and adolescents. Our preliminary findings uncovered significant effects of chrononutrition on pediatric mental health, particularly in adolescents.

The most understudied component of meal timing is the eating window time. Currently, only a handful of studies have examined general eating window time in children and adolescents with high heterogeneity [38]. Therefore, the eating window in pediatric health merits scrutiny. The median eating window time in our study is 11.5 hours, which is similar to previous studies on children and adolescents (11.3 h) [38]. Allied with previous studies that suggest restricting eating window time to 12 hours [29] and advice that 10 hours may be more applicable and physically beneficial [26,39], our results illustrate the occurrence of depression is significantly elevated when the eating duration exceeds 12 hours or is shorter than 10 hours. The lowest depression incidence is observed at 11.5 hours daily eating window.

Time-restricted eating or feeding, commonly limiting the daily intake window to less than 12 hours without restricting total calorie intake [29], is probably the most studied topic related to eating window time due to its promising benefits in promoting metabolic biomarkers and reducing breast cancer risk [18,39]. Thus, some researchers have advocated longer fasting nights and restricted day-eating times. In children and adolescents, time-restricted eating (TRE) is feasible and effective in managing obesity [40], implying the potential to improve other health outcomes as well. The proposed mechanism involves the metabolism of the ketone body in the brain during fast, which upregulates the brain-derived neurotrophic factors and promotes neuroplasticity [41]. In addition, TRE prevented the elevation of neuroinflammation markers in the brain areas involving mood regulation, reducing the risk of depression and anxiety-like behaviors in an animal model [42].

Nevertheless, most studies examining TRE and mental health are targeting adult populations with controversial findings [26,37,42]. A study of 883 older Italian adults examined the relationship between meal timing and cognitive performance. Daily eating duration of less than 10 hours was categorized as the TRE group. TRE and breakfast consumption significantly lowered the risk of cognitive impairment [43]. Conversely, another study on 1572 Italian older adults only found a significant association between TRE (8 h or less) and mental health outcomes, including depressive symptoms, perceived stress, and signs of mental distress [43]. In the Chinese population of 1353 older adults, TRE of less than 10 hours has been associated with impaired cognitive function, particularly the orientation and attention and calculation functions [26]. Yet, more pediatric studies regarding TRE and mental health are warranted due to the distinct physiological and psychological characteristics between the adult and pediatric populations.

Moreover, we found that the omission of breakfast is associated with an approximately 3 times higher risk of depression in children and adolescents. Breakfast is the first meal following the longest fasting period of the day, playing a key role in modifying the peripheral clock [44]. The physical health benefits of breakfast consumption in children and adolescents are concrete. Omission of breakfast is associated with obesity and overweight in children [45], while consuming breakfast ensures nutrition adequacy [46]. Psychologically, skipping breakfast is associated with a significant increase in depression risk [31], the result of which is consistent with our study. Mechanisms underlying the benefits of breakfast remained uncertain. Some proposed theories suggest that breakfast consumption reduces the risk of psychological disorders by decreasing the risk of obesity [31]. Additionally, food groups commonly consumed in breakfast, such as grains, dairy products, and eggs, are rich in micronutrients that are involved in essential neuron functioning [31].

Eating before bed is another aspect of meal timing. In our study population, more than 60% of students reported consuming food 2 hours before bed. The depression odds are 1.28 times higher in people who eat 2 hours before bed. In line with a previous study on university students, delayed eating, as a result of food intake before bed, is associated with a substantially higher risk of mental health conditions [32]. Eating proximal to bedtime has been linked to the accumulation of body fat [17]. In children and adolescents, late and infrequent meal timing has been reported to be associated with insulin-resistant risk [20]. The speculation of the relationship between delayed eating time and depression is rational since obesity and diabetes are commonly comorbid with depression. Additionally, eating at 3 hours before sleep raised the incidence of nocturnal awakening by 40% in a previous study [47]. Disturbance in sleep has long been linked to depression [48], underlying the influence of eating before bed on mental health.

Meal intake is responsible for regulating the circadian rhythms of the peripheral clocks of the human body. The elevation of insulin secretion after food ingestion induces the clock gene regulation pathways, such as PI3/AKT [44]. In addition, food intake may modulate the gastrointestinal system, such as gut microbiota and enteroendocrine cells, signaling the central nervous system via the gut-brain axis [49,50]. In children and adolescents, dietary habits also play as key determinants of sleep quality and circadian sleep-awake cycles by regulating serotonin and melatonin secretions [51]. Disruption of the circadian rhythms, as a result of irregular meal timing, impairs mental health by decreasing neuron complexity and executive function [22]. Thus, proper meal timing is pivotal in determining mental health.

Different from the previous studies, we examined the children and adolescent population with a much larger sample size. Unlike older adults, children and adolescents are at a critical period of physiological and psychological maturation. Coping and emotional regulation skills are essential and intervenable aspects in childhood and adolescence [52]. Therefore, the influence of meal timing on mental health in children and adolescents is more prominent and less confounded by age-related physiological and psychological conditions. Moreover, adolescence, a stage with transformative biological and cognitive growth [41], is subject to dramatic changes in hormones and neurotransmitters [53]. In a critical stage of brain development and psychological maturation [54], adolescents demand substantial dietary support and are more vulnerable to dietary influence [55]. The more prominent effect of meal timing on adolescents than children found in our study is in line with the previous evidence, showing adolescence is a high-risk stage of depression onset [3].

Research on chrononutrition and psychiatry is still in its infancy. Although the revealed relationship in our study may be bidirectional, as a previous study discovered reverse causality between diet and depression [56], findings of our research add more information and aspects in the field of chrononutrition. On the other hand, we uncovered the different effects of meal timing on mental health between children and adolescents. Such differences highlighted the need to develop more stratified dietary and mental health interventions for different age groups. Additionally, schools are pivotal for shaping students’ behaviors and may integrate our findings into school policies to promote healthy dietary behaviors. Furthermore, information generated from this study may guide parents to support and encourage their children to engage in healthy eating practices and lower the risk of depression. Moreover, this research provides a novel dietary aspect to discriminate at-risk groups of depression and promotes the appropriate allocation of health resources.

### Strengths and Limitations

To our knowledge, this study is the first study examining multiple aspects of meal timing and mental health in children and adolescents. We recruited children and adolescents via multistage clustering sampling and yielded a large representative sample. In our analysis, several potential covariables were encompassed, lowering possible confounding effects. Furthermore, we investigated multiple components of chrononutrition, including breakfast consumption, eating proximal to bedtime, and daily eating window. Nevertheless, several limitations need to be noted when interpreting this study. The self-reported dietary data is subject to recall bias. In addition, children and adolescents are a highly versatile group whose health is influenced by school, peers, geography, culture, and family. Therefore, the optimal eating duration may not be generalized to other unanalogous populations. The cross-sectional design of this research limits the establishment of causal relationships. While this study suggests a link between meal timing and depression, further longitudinal research is needed to establish a causal relationship and determine the long-term effects. Conducting longitudinal studies that follow children and adolescents over an extended period can provide stronger evidence and help identify other factors that may influence the relationship between chrononutrition and mental health outcomes. Moreover, meal timing on weekdays and weekends was not distinguished in this study, which may be studied separately in future studies.

### Conclusions

Breakfast skipping and eating before bed increases the odds of depression by 2.7 and 1.28 times. Furthermore, daily eating windows longer than 12 hours and shorter than 10 hours substantially raised the occurrence of depression by 1.23 and 1.37 times, respectively. Current findings advocate evidence-based strategies to implement prevention and treatment of depression in children and adolescents.

## Supplementary material

10.2196/54275 Multimedia Appendix 1 Subgroup analyses stratified by age (children <10 years, adolescents 10-19 years).

## Abbreviations

OR: odds ratio
TRE: time-restricted eating
