# Correction: Meal Timing and Depression Among Chinese Children and Adolescents: Cross-Sectional Study

**DOI:** 10.2196/73716

**Published:** 2025-04-02

**Authors:** Huilun Li, Zhaohui Lu, Erliang Zhang, Jie Zhang, Shuheng Cui, Masaki Takahashi, Mi Xiang

**Affiliations:** 1 Hainan Branch, Shanghai Children’s Medical Center, School of Medicine, Shanghai Jiao Tong University, Sanya, China; 2 School of Public Health, Shanghai Jiao Tong University, 227 S Chongqing Rd, Shanghai, 200025, China; 3 Institute for Liberal Arts, Tokyo Institute of Technology, Tokyo, Japan

In “Meal Timing and Depression Among Chinese Children and Adolescents: Cross-Sectional Study” (JMIR Public Health Surveill 2024;10:e54275) the authors noted one error.

The funding information was absent from the final article, which has been added to the Acknowledgements section as follows:

*This work was supported by The National Natural Science Foundation of China (No. 71804110), Shanghai Science and Technology Development Funds (No. 21QA1405300), Science Foundation for new teachers of Shanghai Jiao Tong University School of Medicine (No. 20X 100040012), Science Foundation of Ministry of Education of China (No.22YJAZH116), and Shanghai Municipal Health Commission (No.: GW-10.1-XK07*).

The correction will appear in the online version of the paper on the JMIR Publications website, together with the publication of this correction notice. Because this was made after submission to PubMed, PubMed Central, and other full-text repositories, the corrected article has also been resubmitted to those repositories.

