# Supplementary material for: Meal Timing and Depression Among Chinese Children and Adolescents: Cross-Sectional Study
Source: JMIR Public Health Surveill. 2024 Oct 23;10:e54275. doi: 10.2196/54275 (PMC11615707; doi:10.2196/54275)
Supplement: Multimedia Appendix 1 [file publichealth-v10-e54275-s001.docx]

Supplementary table 1. Frequencies of breakfast consumption, eating before bed, and daily eating window time variables according to depression incidence: subgroup analysis stratified by age (children <10 years, adolescents 10-19 years)

|  | Overall | Population | | *P* value |
| --- | --- | --- | --- | --- |
| Variables |  | Children  (n=3548) | Adolescents  (n=3326) |  |
| **Breakfast consumption^b^, n%** |  |  |  | <.001 |
| Consuming breakfast | 6106 (88.8%) | 3276 (92.3%) | 2830 (85.1%) |  |
| Skipping breakfast | 768 (11.2%) | 272 (7.7%) | 496 (14.9%) |  |
| **Eating before bed^c^, n%** |  |  |  | .046 |
| No | 2376 (34.6%) | 1187 (33.5%) | 1189 (35.7%) |  |
| Yes | 4498 (65.4%) | 2361 (66.5%) | 2137 (64.3%) |  |
| **Daily eating window (hours)^d^, median (Q_1_, Q_3_)** | 11.5 (10.5, 11.5) | 10.5 (10.5,11.5) | 11.5 (10.5,11.5) | <.001 |

Supplementary table 2. Logistic regression analyses of breakfast consumption, eating before bed, and daily eating window in relation to depression incidence: subgroup analysis stratified by age (children <10 years, adolescents 10-19 years)

|  | Unadjusted model  OR(95%CI) | *P* value | Model 2^a^  OR(95%CI) | *P* value | Model 3^b^  OR(95%CI) | *P* value |
| --- | --- | --- | --- | --- | --- | --- |
| **Children (n=3548)** |  |  |  |  |  |  |
| **Breakfast consumption^c^** |  |  |  |  |  |  |
| Consuming breakfast | Ref. |  | Ref. |  | Ref. |  |
| Skipping breakfast | 3.02 (2.15-4.25) | <.001 | 2.77 (1.95-3.93) | <.001 | 2.77 (1.94-3.96) | <.001 |
| **Eating before bed^d^** |  |  |  |  |  |  |
| No | Ref. |  | Ref. |  | Ref. |  |
| Yes | 1.11 (0.85-1.46) |  | 1.18 (0.896-1.55) | .24 | 1.11 (0.84-1.47) | .46 |
| **Eating window time (hours)^e^** |  |  |  |  |  |  |
| 10 to 12 | Ref. |  | Ref. |  | Ref. |  |
| ≤ 10 | 1.33 (0.92-1.91) | .13 | 0..82 (0.52-1.28) | .37 | 1.30 (0.90-1.90) | .17 |
| ≥ 12 | 1.34 (0.87-2.07) | .19 | 1.12 (0.65-1.92) | .69 | 1.13 (0.72-1.77) | .60 |
| **Adolescents (n=3326)** |  |  |  |  |  |  |
| **Breakfast consumption^c^** |  |  |  |  |  |  |
| Consuming breakfast | Ref. |  | Ref. |  | Ref. |  |
| Skipping breakfast | 3.33 (2.70-4.12) | <.001 | 2.87 (2.31-3.57) | <.001 | 2.69 (2.16-3.35) | <.001 |
| **Eating before bed^d^** |  |  |  |  |  |  |
| No | Ref. |  | Ref. |  | Ref. |  |
| Yes | 1.51 (1.25-1.84) | <.001 | 1.52 (1.25-1.86) | <.001 | 1.38 (1.12-1.69) | .002 |
| **Eating window time (hours)^e^** |  |  |  |  |  |  |
| 10 to 12 | Ref. |  | Ref. |  | Ref. |  |
| ≤ 10 | 1.70 (1.27-2.26) | <.001 | 0.75 (0.61-0.94) | .011 | 1.41 (1.04-1.91) | .026 |
| ≥ 12 | 1.49(1.20-1.84) | <.001 | 1.18 (0.85-1.64) | .33 | 1.27 (1.01-1.58) | .038 |

^a^ Model 2 adjusted for age, sex, BMI, parental education level, and family income

^b^ Model 3 adjusted for caloric intake and other aspects of meal timing

^c^ Breakfast consumption: participants who skipped breakfast at least once per week are categorized into the skipping breakfast group

^d^ Eating before bed: participants eat one or more times before bed are classified as the eating-before-bed group.

^e^ Daily eating window: last food intake time frame - first food intake time frame
